# Muscle-specific kinase levels in blood are an early diagnostic biomarker for SOD1-93A mouse model of ALS

**DOI:** 10.3389/fneur.2025.1556120

**Published:** 2025-04-28

**Authors:** Shuuichi Mori, Heying Zhou, Takuya Omura, Hiroki Tsumoto, Yuri Miura, Kazuhiro Shigemoto

**Affiliations:** ^1^Research Team for Geriatric Medicine, Tokyo Metropolitan Institute for Geriatrics and Gerontology, Tokyo, Japan; ^2^Research Team for Mechanism of Aging, Tokyo Metropolitan Institute for Geriatrics and Gerontology, Tokyo, Japan

**Keywords:** SOD1 mouse G93A, NMJ, biomarker, MuSK, diagnosis, mouse model

## Abstract

Neuromuscular junction (NMJ) denervation is an early event preceding motor neuron loss in amyotrophic lateral sclerosis (ALS). Progressive loss of the NMJ leads to irreversible muscle weakness and atrophy. Muscle-specific kinase (MuSK), locally expressed at the postsynaptic membrane of the NMJ, is activated by agrin released from motor nerve terminals and is essential for NMJ maintenance and regeneration. Here, we found that the progression of NMJ denervation prior to the onset of muscle weakness in SOD1-93A mouse model of ALS correlated with increased serum MuSK immunoreactivity and elevated MuSK expression throughout the skeletal muscle. Our results suggest that neuromuscular failure associated with the onset of muscle weakness increases MuSK expression throughout the muscle, which is subsequently cleaved by proteolytic enzymes to increase MuSK immunoreactivity in the blood. These results demonstrate that the level of serum MuSK immunoreactivity may indicate the early phase of NMJ denervation and serve as a biomarker for assessing the progression of other types of ALS and therapeutic benefits in preclinical studies.

## Introduction

Amyotrophic lateral sclerosis (ALS) is a devastating neurodegenerative disease that progresses from a subtle decline in motor function to fatal respiratory paralysis within years of diagnosis. Most cases of ALS are considered sporadic (sALS), in which both genetic and environmental factors contribute to the pathogenesis, whereas 10% of patients with ALS have a family history of the disease (i.e., familial ALS) (1, 2). Approximately 2% of patients with ALS have mutations in the superoxide dismutase 1 (SOD1) gene, and transgenic mice with SOD1-93A mutations have been shown to develop progressive weakness similar to that observed in patients with ALS (3). Therefore, these mice serve as a useful model for understanding the pathogenesis of ALS-like phenotypes and testing new therapies. Weakness and death in SOD1-93A mice occur directly as a consequence of motor neuron death; however, neuromuscular junction (NMJ) denervation occurs more earlier than motor neuron loss (4–9). Biopsy samples from patients with sALS have also shown significantly higher levels of partial and complete NMJ denervation compared to those in controls (8), and muscle weakness and wasting appear with the progression of denervation.

Muscle-specific kinase (MuSK) proteins are receptor-type tyrosine kinases localized and expressed in the postsynaptic membrane of the NMJ (10). MuSK is activated by agrin, a heparin sulfate proteoglycan released from nerve terminals, and low-density lipoprotein receptor (LDLR)-related protein 4 (LRP4) acts as a coreceptor (11–13). MuSK phosphorylation is required for the anchoring and clustering of acetylcholine receptors (AChRs) to the postsynaptic membrane of the NMJ via Dok7, an essential intracellular MuSK-binding protein (14, 15). Furthermore, MuSK is required for the maintenance of both pre- and postsynaptic functions and morphology (16). We recently evaluated the extracellular domain of MuSK in human and mouse serum and found that neuromuscular transmission failure increased the shedding of the MuSK ectodomain by metalloproteinases in skeletal muscle (17). In a mouse model of motor nerve crush injury, levels of serum MuSK immunoreactivity increased following denervation and returned to normal after reinnervation. Multiple lines of evidence, including muscle biopsies, post-mortem studies, and electrophysiological tests, have demonstrated that neuromuscular junction (NMJ) dysfunction is a key contributor to the early stages of ALS progression (4, 9). Therefore, in this study, based on the hypothesis that blood MuSK immunoreactivity is elevated in SOD1-93A mice prior to the onset of weakness after denervation, we analysed whether MuSK immunoreactivity levels in the blood increased as signs of weakness and weight loss progressed.

## Materials and methods

### Animals

All animal procedures were conducted in accordance with the Basic Animal Care and Experimental Guidelines of the Ministry of Health, Labour and Welfare of Japan and were approved by the Experimental Animal Care and Use Committee of the Tokyo Metropolitan Institute of Gerontology (License No. 16036). Mice overexpressing a human SOD mutant [B6.Cg-Tg (SOD1-G93A) 1Gur/J] were purchased from The Jackson Laboratory (Bar Harbor, ME), and mutant mice were identified through standard polymerase chain reaction (PCR) analysis of tail section DNA. The mutant SOD1 transgene was maintained as a hemizygous trait by breeding hemizygous males with wild-type females (C57BL/6). The C57BL/6 mice were obtained from Japan SLC (Hamamatsu, Japan). Due to sex-related differences in lifespan, muscle size, and muscle strength in SOD1 mice, only male mice were used in the experiments to avoid ambiguity caused by sex. Mice used in the experiments were euthanised by cervical dislocation without anaesthesia, which was approved under the license (No. 16036). We made every effort to minimize animal suffering during the course of our study, adhering strictly to ethical guidelines and approved protocols.

### Production of recombinant MuSK and MuSK monoclonal antibodies

Recombinant mouse and human MuSK ectodomain proteins and MuSK monoclonal antibodies (mAbs) were prepared as previously described (17). Mouse MuSK ectodomain DNA fragment was PCR-amplified from differentiated C2C12 cell template cDNA using primer set 5′-CGGAATTCCAGAAGCAACCTTTCTTCCTGAGC-3′ (forward) and 5′-TCCTCTAGATTAGTGATGGTGATGGTGATGACTTCCAAAGTCTGGAGGAACTTCTTT-3′ (reverse) (AY360453; GenBank). The PCR fragment was inserted into the EcoRI and Xho sites of the pSV-SPORT vector (Life Technologies). The DNA fragment of the human MuSK ectodomain was amplified by PCR using the full-length human MuSK cDNA isolated in our previous study (17) as a template with the primer set 5′-GGAATTCACTTCGTCCTGCGTGAGCCT-3′ (forward) and 5′-CCGCTCGAGCATGGAGTATGTAGGTGAGAC-3′ (reverse) (AF006464; GenBank). The PCR fragment was inserted into the EcoRI and Xho sites of the pCDNA 3.1 vector (Invitrogen). Expression vectors were transfected into human embryonic kidney 293-F cells using the FreeStyle MAX reagent (Life Technologies, United States), and hexahistidine-tagged proteins secreted into the culture medium were purified using Ni-Sepharose (17-5318-02; GE Healthcare, United States).

Hybridomas were generated by fusing myeloma cells with splenocytes from rats immunised with the mouse MuSK protein (Kurabo, Japan) and from mice and rabbits immunised with the human MuSK protein (Kurabo, Japan, and Epitomics, United States, respectively). Previously reported MH-18 (mouse mAb), MH-30 (mouse mAb), RM-24 (rat mAb), and RbH-2 mAbs (rabbit mAb) were used in the experiments (17). The mAb was purified from the hybridoma supernatant using a HiTrap Protein G column (GE Healthcare, United States). The monoclonal antibodies used in this study were used at the same dilution concentrations reported previously (17).

### Amplified luminescent homogeneous immunoassay of MuSK

The amplified luminescent homogeneous immunoassay (AlphaLISA, PerkinElmer, United States) was performed to quantify serum MuSK concentrations using purified mouse anti-MuSK antibody mAb (MH-30) and rat anti-MuSK mAb (RM-24), as described previously (17). Serum samples were collected at 6, 10, and 20 weeks of age.

### Western blotting

Western blot detection of the immunoprecipitated MuSK protein was performed on protein lysates extracted from the gastrocnemius muscles using mouse anti-MuSK mAbs (MH-30 and MH-18) and rabbit anti-MuSK mAb (RbH-2), as described previously (17). Mouse gastrocnemius muscles were harvested at 6, 10, and 20 weeks of age. Protein extracts were prepared using protein lysis buffer and protease inhibitors (Roche, Basel, Switzerland). Sodium dodecyl sulfate-polyacrylamide gel electrophoresis was performed, and the proteins were transferred to polyvinylidene difluoride membranes. The signals were detected using IRDye800-conjugated goat anti-rabbit immunoglobulin G (IgG) antibodies (LI-COR Biosciences, United States). Images and band intensities were captured and recorded using an Odyssey Infrared Imaging System (LI-COR Biosciences, United States).

### Immunofluorescence staining

Immunofluorescence staining and image analysis of NMJs were performed as previously described (17). Briefly, 40-μm-thick longitudinal sections were initially stained with rhodamine-conjugated α-bungarotoxin (BTx; Life Technologies, United States). The sections were washed, permeabilized with ice-cold methanol, and incubated with rabbit anti-synaptophysin (Life Technologies, United States). After washing with phosphate-buffered saline, the sections were incubated with Alexa Fluor 488-labeled goat anti-rabbit IgG (Life Technologies). The 20-μm-thick longitudinal sections were stained with rabbit anti-MuSK (RbH-8) mAb. Images were acquired using a Leica TCS SP8 confocal microscope (Leica Microsystems, Germany) with a 20× objective lens (for synaptophysin staining) or a 63× objective lens (for MuSK staining). Innervation was evaluated on acquired images based on the following criteria. The scores were divided into three categories: fully occupied (almost complete overlap of staining between endplates and nerve endings), partially occupied (slight overlap of staining between endplates and nerve endings), and unoccupied (almost no overlap between endplates and nerve endings). Using ImageJ software (Version 1.42q; National Institutes of Health, United States), we defined region of interest (ROIs) for NMJs and synaptic membranes and quantified the mean intensity of MuSK staining within them. The MuSK-stained area of the extrasynaptic membrane was defined as the entire fascia outside the BTx-stained area on each image.

### Quantitative PCR

This experiment was conducted as previously described (17). Total RNA was isolated from the gastrocnemius muscle using TRIzol reagent (Life Technologies, United States) and subjected to reverse transcription using the GoScript Reverse Transcription System (Promega, United States). The mRNA abundance was normalized to that of glyceraldehyde 3-phosphate dehydrogenase. Real-time PCR analysis was performed in duplicate using GoTaq qPCR Master Mix (Promega, United States). PCR reactions were carried out using the following primer sets:

MUSK, 5′-CTCGTCCTCCCATTAATGTAAAAA-3′ (forward) and 5′-TCCAGCTTCACCAG-TTTGGAGTAA-3′ (reverse).
GAPDH, 5′-CCATCACCATCTTCCAGGAG-3′ (forward) and 5′-GTGGTTCACACCCATCACAA-3′ (reverse).

### Behavior test

The wire-hanging test was conducted as described previously (17, 18). Briefly, the mice were placed on the wire mesh of a wire-cage lid apparatus (O’Hara & Co., Ltd.), gently inverted, and held on a soft surface. The mice made up to three attempts to cling to the inverted lid for a period of up to 90 s, and the longest time was recorded.

### Statistical analyses

Data were presented as the mean ± standard deviation. All statistical analyses were performed using Microsoft Excel and Prism 10 (GraphPad Software, United States). Differences between the two groups were assessed for statistical significance using an unpaired *t*-test. The significance of time-series group comparisons was evaluated using a two-way analysis of variance, followed by Sidak’s multiple-comparisons test. Statistical significance was set at *p* < 0.05.

## Results

### Serum MuSK levels increased before onset of muscle weakness in SOD1-93A mice

**Figure 1:**
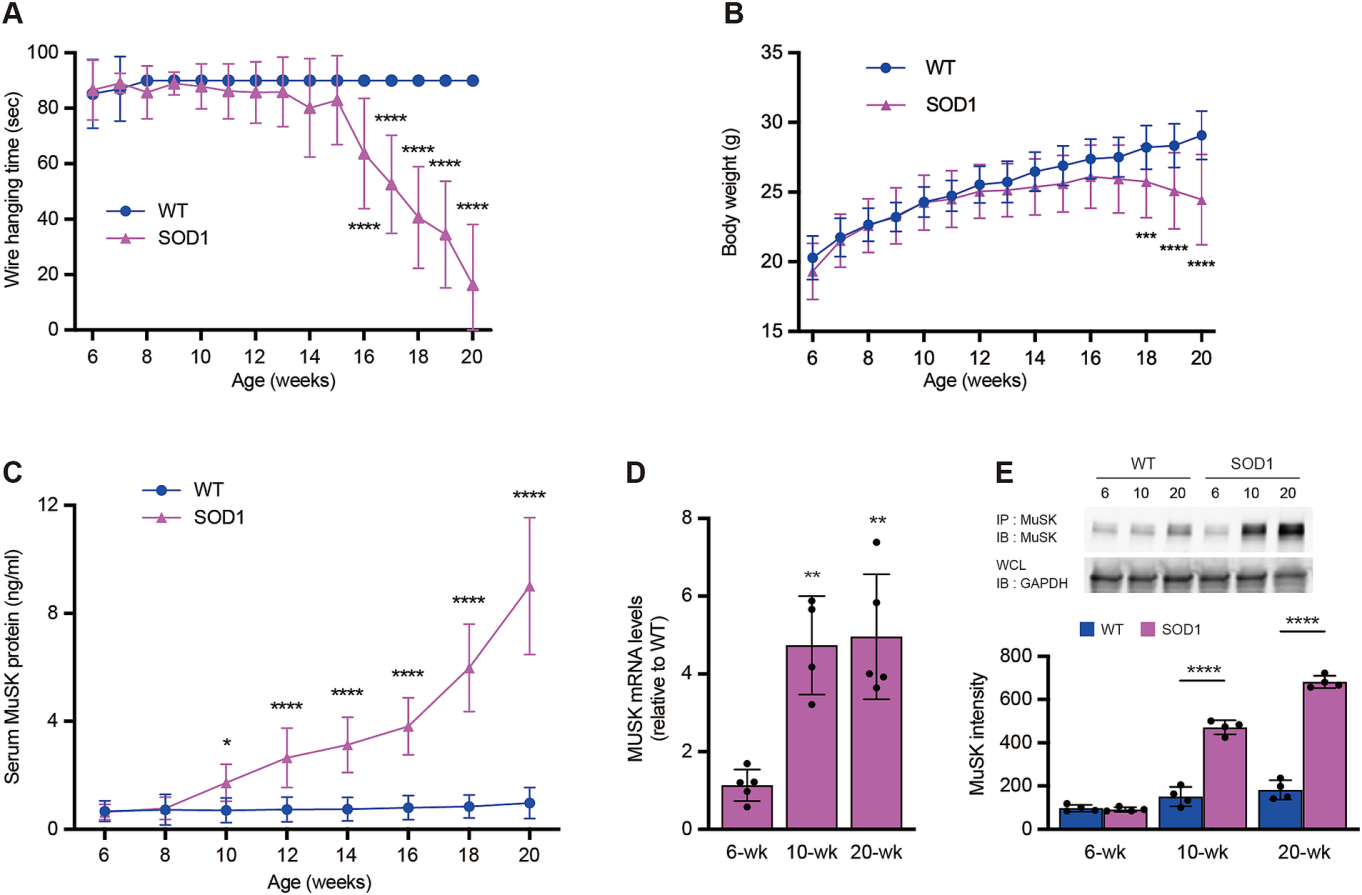
Serum muscle-specific kinase levels reflect the progression of amyotrophic lateral sclerosis in SOD1 mice. Changes in wire-hanging time **(A)**, body weight **(B)**, and serum MuSK immunoreactivity levels **(C)** in SOD1 mice and their WT littermates (*n* = 15; ^*^p < 0.05, ^**^p < 0.01, ^***^p < 0.001, and ^****^p < 0.0001 vs. WT mice at the same time point). Evaluation of MuSK mRNA expression **(D)** and protein levels **(E)** (*n* = 4–6); gastrocnemius muscles were harvested at 6, 10, and 20 weeks of age for RNA and protein extraction. mRNA levels are shown as a ratio relative to those in WT mice at each time point. WT, wild-type; GAPDH, glyceraldehyde 3-phosphate dehydrogenase.

We examined muscle strength, weight loss, and serum MuSK immunoreactivity levels in normal and SOD1-93A mice. In addition to the fact that the rate of progression of muscle weakness and weight loss in SOD1-93A mice varies depending on the rearing environment, this is the first study to measure and compare changes in MuSK immunoreactivity in the blood of the same mice in parallel with decreases in body weight and grip strength. Muscle strength measurements in the wire suspension test showed that the first significant muscle weakness was observable at 16 weeks of age (Figure 1A), whereas body weight decreased significantly at 18 weeks of age (Figure 1B). However, serum MuSK immunoreactivity levels increased significantly from 10 weeks of age, much earlier than the onset of muscle weakness and weight loss (Figure 1C). Serum MuSK immunoreactivity levels increased significantly by 14.2-fold during the observation period from 6 to 20 weeks of age. MuSK mRNA (Figure 1D) and protein expression (Figure 1E) levels in the gastrocnemius muscle of SOD1-93A mice increased significantly from 10 weeks of age and correlated with elevated serum MuSK immunoreactivity levels. Therefore, our results show that serum MuSK immunoreactivity levels are elevated even before the onset of weakness in ALS mice.

### Serum MuSK immunoreactivity levels are associated with neuromuscular denervation in SOD1-93A mice

**Figure 2:**
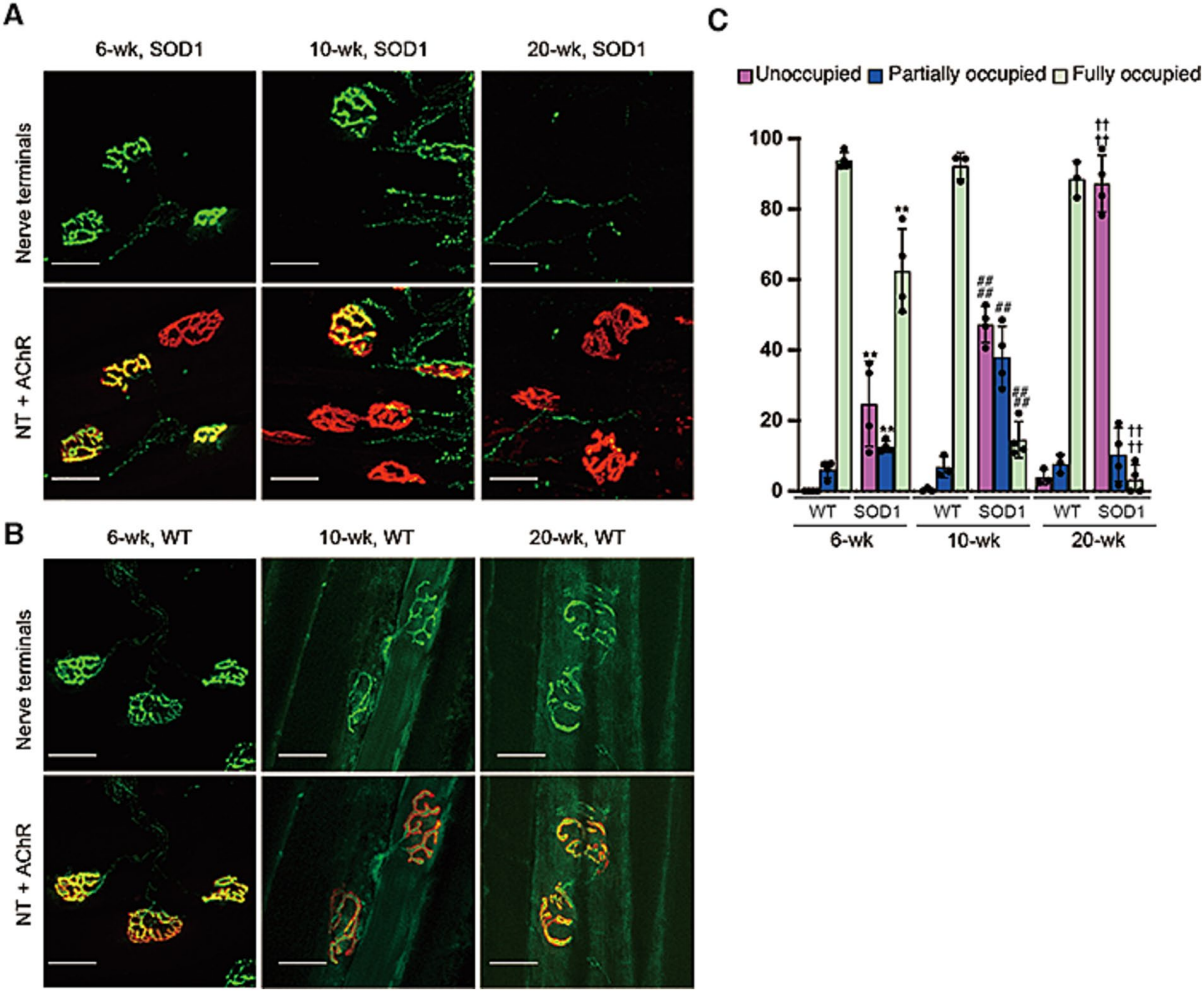
Progression of NMJ denervation with increased serum MuSK immunoreactivity levels. Representative immunofluorescence staining **(A,B)** and quantification **(C)** of nerve terminals [(NT), green] and rhodamine-conjugated α-bungarotoxin [acetylcholine receptors (AChRs), red] at the NMJ. Longitudinal sections of tibialis anterior muscles were obtained from SOD1-93A **(A)** and normal mice **(B)** at 6, 10, and 20 weeks of age. Scale bar: 30 μm. Images of 91–203 NMJs were acquired from each mouse. Data are shown as the percentage of endplates classified as fully occupied, unoccupied, or partially occupied (*n* = 3–4; ^**^p < 0.01 vs. wild-type mice at 6 weeks of age; ^##^*p* < 0.01 and ^####^*p* < 0.0001 vs. SOD1 mice at 10 weeks of age, ^††††^*p* < 0.0001 vs. SOD1 mice at 20 weeks of age).

Next, we investigated the temporal relationship between elevated blood MuSK immunoreactivity levels and NMJ denervation in SOD1-93A mice. NMJ denervation in SOD1-93A mice has been reported to occur before disease onset (7). The co-localization of presynaptic and postsynaptic structures in the NMJ was examined by histochemical analysis of SOD1-93A and normal mice at 6, 10 and 20 weeks of age, and the findings were compared across these time points (Figures 2A,B). Immunofluorescence staining of the anterior tibialis muscle of SOD1-93A mice from 6 to 20 weeks of age showed progressive denervation of nerve terminals with increasing disease severity. The levels of both fully unoccupied and partially occupied endplates were significantly greater (*p* < 0.01) than those in wild-type mice 10 weeks before onset of muscle weakness and 4 weeks before elevated blood MuSK levels (Figures 2A–C). By 20 weeks, approximately 87.3% of the nerve terminals were completely denervated (Figures 2A,B). Taken together, our data indicated that serum MuSK immunoreactivity levels in SOD1-93A mice indirectly reflected NMJ denervation during disease progression.

### Altered localization and expression of MuSK in SOD1-93A mice

**Figure 3:**
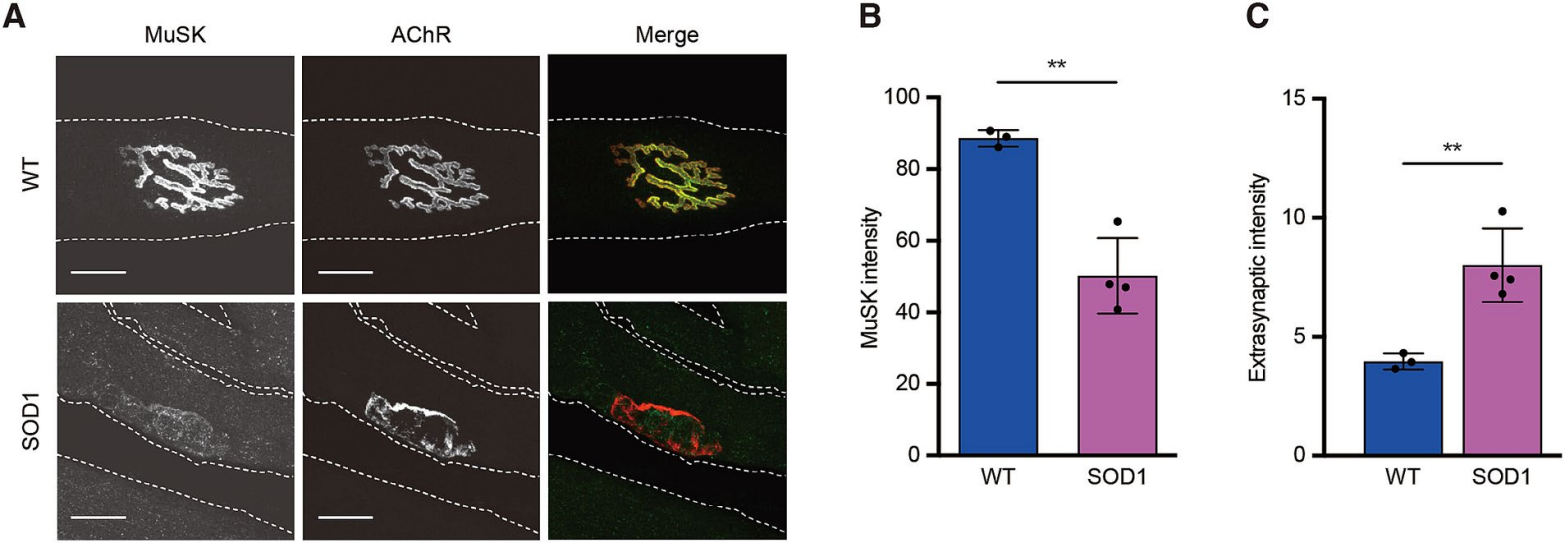
Localization and expression of muscle-specific kinase in the motor endplate of neuromuscular junctions and muscles. Representative immunofluorescence staining **(A)** and quantification of MuSK at neuromuscular junctions **(B)** and extrasynaptic membranes **(C)**; AChRs, acetylcholine receptors. Longitudinal sections of tibialis anterior muscles were harvested from 20-week-old mice. Myofiber boundaries are indicated by dashed lines. Scale bar: 20 μm. Any region of the muscle membrane outside the rhodamine-conjugated α-bungarotoxin-stained area in the acquired image was selected as the extrasynaptic membrane. Images of 41–72 neuromuscular junctions were acquired from each mouse (*n* = 3–4, ^**^p < 0.01). Data represent the mean ± standard deviation.

In a previous study, MuSK expression at the endplates of the extensor digitorum longus muscle in SOD1-93A mice was shown to decrease with denervation (19). Patients with sALS have also been reported to exhibit decreased MuSK immunostaining in the motor endplate of the vastus lateralis muscle (8). In contrast, MuSK expression in the motor endplate of the tibialis anterior muscle remained unchanged compared with that in normal mice in the mouse nerve transection model (17). Therefore, we performed immunostaining to examine the localization and expression of AChRs and MuSK in the motor endplates of the tibialis anterior muscles of SOD1-93A mice. The mean intensity of MuSK fluorescence in the endplates of SOD1-93A mice at week 20 was significantly lower (*p* < 0.01) (Figures 3A,B), whereas the mean intensity of MuSK fluorescence outside the synaptic region was significantly higher (*p* < 0.01) (Figure 3C), as observed in a mouse nerve transection model (Figures 3A,B) (17). The reduced MuSK expression in the NMJ is consistent with results reported in previous studies of patients with sALS and SOD1-93A mice (8, 17), whereas extrasynaptic MuSK expression was strongly enhanced in SOD1-93A mice, as observed in the mouse nerve transaction model (17). As in previous studies, there was no significant difference in the staining of rhodamine-conjugated α-bungarotoxin in the muscles of SOD1-93A and wild-type mice (Figure 3A) (19, 20).

## Discussion

Recent preclinical studies have shown that therapies targeting MuSK signalling in the NMJ slow the onset and extend the lifespan of SOD1-93A mice (21–25). Such preclinical studies require the objective and reliable quantification of disease progression in individual mice. This study provided the first evidence that serum MuSK levels in SOD1-93A male mice increase from the asymptomatic phase as NMJ denervation progresses. The pathological features of ALS-like phenotypes are well reproduced in mice overexpressing the human SOD1 mutant (3, 4, 7, 8). Our results suggest that blood MuSK immunoreactivity levels can serve as biomarkers for NMJ dysfunction prior to the onset of weakness in this ALS model. We have previously shown that when the sciatic nerve of mice is crushed, serum MuSK immunoreactivity levels initially increase and then return to normal as NMJs regenerate. In contrast, nerve transection, which prevents NMJs regeneration, irreversibly increases blood MuSK immunoreactivity levels (17). We have also reported elevated blood MuSK immunoreactivity levels in patients and in a mouse model of myasthenia gravis (17), a disease characterised by impaired neurotransmission due to autoantibodies against NMJs. Other mouse models of severe ALS with human TAR DNA-binding protein-43 or fused-in-sarcoma mutations also exhibit early NMJ pathology, similar to that observed in SOD1 mice (9, 26, 27). Studies on the NMJs in patients with ALS are challenging due to difficulties in obtaining samples; adequately controlling for age, sex, and disease duration; correlations with medical and family history; and the inability to obtain samples prior to symptom onset (9). However, a combination of muscle biopsy evaluations, post-mortem analysis, and electrophysiological assessments has shown that NMJ dysfunction plays a significant role in the early progression of ALS (4, 9). Future studies should aim to determine whether serum MuSK immunoreactivity levels in other ALS models and patients with ALS can serve as early biomarkers for assessing neuromuscular dysfunction prior to motor neuron death.

Our previous studies using nerve crush and transection models in mice showed that two mechanisms are involved in the release of MuSK into the circulation: increased MuSK gene expression after NMJ denervation and ectodomain shedding of MuSK proteins by metalloproteases (17). Myogenin, a transcription factor belonging to the basic helix-loop-helix family, plays a crucial role in controlling both the expression levels and spatial distribution of MuSK in muscle tissue. It binds to and activates E-box sequences located within the MuSK gene promoter (28). In healthy, innervated muscles, the transcription of myogenin is actively suppressed, limiting MuSK production to a few nuclei positioned beneath the NMJs. The initiation of myogenin expression is dependent on the function of histone deacetylases (HDACs) (28–30). Among these, HDAC4 is notably concentrated in the nuclei directly beneath innervated NMJs, while its expression is minimal in the nuclei of non-synaptic muscle regions. However, in response to reduced neural input—caused by nerve damage or neuromuscular disorders—HDAC4 expression is upregulated and accumulates in nuclei beyond the synaptic zone. This increase in HDAC4 activity leads to a reduction in Dach2, a transcriptional co-repressor that normally inhibits myogenin. As a result, myogenin expression is enhanced in these non-synaptic regions, triggering MuSK production outside the NMJs. When neural stimulation is re-established, both myogenin and MuSK expression levels return to their original, localized patterns. Similar to the nerve transection model, SOD1-93A mice exhibited increased MuSK expression in skeletal muscle after denervation, with a corresponding increase in blood MuSK levels, suggesting that extrasynaptic MuSK is the primary source of serum MuSK protein. The mouse nerve transection model has been shown to exhibit similar levels of MuSK expression in the postsynaptic region of the NMJ as those observed in normal mice, whereas MuSK expression is significantly reduced in the postsynaptic region of the NMJ in both SOD1-93A mice and patients with sALS. The decrease in MuSK levels at the endplates of SOD1-93A mice may result from defective MuSK recycling in skeletal muscles, as reported in a previous study (19). However, future studies should aim to determine whether SOD1-93A mice and individuals with ALS share a common mechanism for the reduced expression of MuSK at the NMJ.

The second mechanism underlying the release of MuSK into the blood involves the proteolytic cleavage of elevated extrasynaptic MuSK by metalloproteinases (MMPs) (17). The ectodomains of numerous membrane proteins are proteolytically cleaved by MMPs and subsequently secreted extracellularly. MMPs are important regulators of various biological functions but are also involved in several pathological processes (31, 32). MMP-1, MMP-2, MMP-3, and MMP-9 levels in the blood and cerebrospinal fluid are reported predictors of ALS progression, with neuronal damage implicated in ALS pathogenesis (33, 34). Mouse models of nerve crush and transection may share a common mechanism with SOD1-93A mice, as the disruption of neurotransmission triggers MMPs to activate MuSK shedding, resulting in increased MuSK immunoreactivity levels in the blood (17). However, the matrix proteases that cleave the extracellular domain of skeletal muscle MuSK have not yet been identified, and their physiological and pathological roles remain to be elucidated.

## Conclusion

The assessment of pre-onset denervation by measuring MuSK immunoreactivity levels in the blood, without pathological analysis, could facilitate the development of effective therapeutic agents.

## Data Availability

The original contributions presented in the study are included in the article/Supplementary material, further inquiries can be directed to the corresponding author.
