# Supplementary figures and images for: Muscle-specific kinase levels in blood are an early diagnostic biomarker for SOD1-93A mouse model of ALS
Source: Front Neurol. 2025 Apr 28;16:1556120. doi: 10.3389/fneur.2025.1556120 (PMC12066615; doi:10.3389/fneur.2025.1556120)

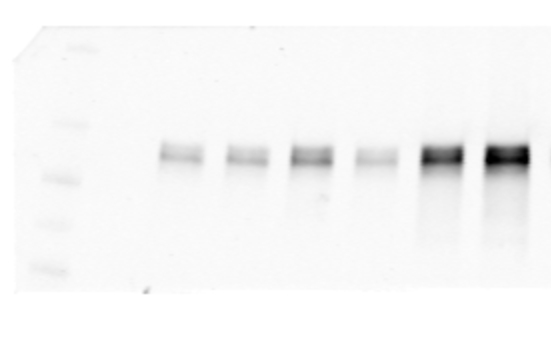

Supplement: Supplementary file 2 [file Image_1.TIFF]

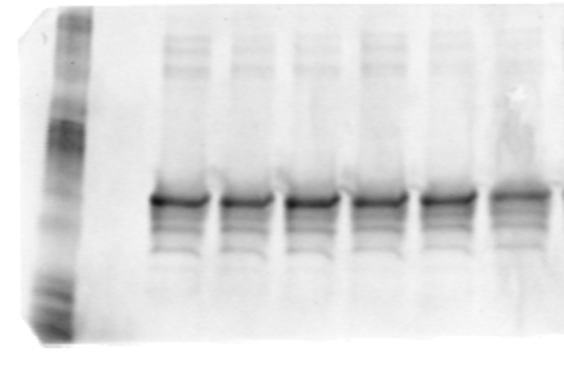

Supplement: Supplementary file 3 [file Image_2.TIF]
